# A novel variant in *FOXC1* associated with atypical Axenfeld-Rieger syndrome

**DOI:** 10.1186/s12920-021-01130-7

**Published:** 2021-11-22

**Authors:** Rui Wang, Wei-Qian Wang, Xiao-Qin Li, Juan Zhao, Kun Yang, Yong Feng, Meng-Meng Guo, Min Liu, Xing Liu, Xi Wang, Yong-Yi Yuan, Xue Gao, Jin-Cao Xu

**Affiliations:** 1 Postgraduate Training Base Of Jinzhou Medical University (The PLA Rocket Force Characteristic Medical Center), 16# XinWai Da Jie, Beijing, 100088 People’s Republic of China; 2 Department of Otolaryngology, The PLA Rocket Force Characteristic Medical Center, 16# XinWai Da Jie, Beijing, 100088 People’s Republic of China; 3 College of Otolaryngology Head and Neck Surgery, Chinese PLA General Hospital, Chinese PLA Medical School, 28 Fuxing Road, Beijing, 100853 People’s Republic of China; 4 National Clinical Research Center for Otolaryngologic Diseases, State Key Lab of Hearing Science, Ministry of Education, China, Beijing Key Lab of Hearing Impairment Prevention and Treatment, Beijing, People’s Republic of China; 5 Department of Ophthalmology, The PLA Rocket Force Characteristic Medical Center, 16# XinWai Da Jie, Beijing, 100088 People’s Republic of China

**Keywords:** *FOXC1*, Whole exome sequencing, Axenfeld-Rieger syndrome

## Abstract

Mutations in the Forkhead Box C1 (*FOXC1*) are known to cause autosomal dominant hereditary Axenfeld-Rieger syndrome, which is a genetic disorder characterized by ocular and systemic features including glaucoma, variable dental defects, craniofacial dysmorphism and hearing loss. Due to late-onset of ocular disorders and lack of typical presentation, clinical diagnosis presents a huge challenge. In this study, we described a pathogenic in-frame variant in *FOXC1* in one 5-year-old boy who is presented with hypertelorism, pupil deformation in both eyes, conductive hearing loss, and dental defects. By whole exome sequencing, we identified a 3 bp deletion in *FOXC1,* c.516_518delGCG (p.Arg173del) as the disease-causing variant, which was de novo and not detected in the parents, and could be classified as a “pathogenic variant” according to the American College of Medical Genetics and Genomics guidelines. After confirmation of this *FOXC1* variant, clinical data on Axenfeld-Rieger syndrome-associated clinical features were collected and analyzed. Furthermore, Although the affected individual present hearing loss, however, the hearing loss is conductive and is reversible during the follow-up, which might not linke to the *FOXC1* variant and is coincidental. Routine examination of *FOXC1* is necessary for the genetic diagnosis of hypertelorism-associated syndrome. These findings may assist clinicians in reaching correct clinical and molecular diagnoses, and providing appropriate genetic counseling.

**Supplementary Information:**

The online version contains supplementary material available at 10.1186/s12920-021-01130-7.

## Introduction

Axenfeld-Rieger syndrome (ARS) [MIM #602482] is an autosomal dominant disorder which is characterized by ocular and systemic features of variable degrees. ARS is a rare disorder, with prevalence estimated at 1 in 50,000 to 100,000 newborns. Patients with ARS present with ocular malformations particularly in the iris, cornea and the chamber angle, which belongs to the anterior segment of the eye. The presence of anomalies in the anterior chamber angle and drainage structure of the eye contributes to lifetime risk of developing glaucoma and can lead to irreversible blindness. Approximately 50% of ARS patients will develop into glaucoma, with an age of onset ranging from birth to late in adulthood [1, 2]. Onset of glaucoma typically occurs before the teenage years [3].

In addition to the ocular features, ARS may have systemic abnormalities including craniofacial hypoplasia, dental and umbilical anomalies, cardiac defects, and hearing loss [4–6]. The most characterized systemic features are mild craniofacial dysmorphism, dental anomalies, and redundancy of periumbilical skin. Craniofacial features can be helpful in suggesting a diagnosis of ARS, particularly in family members with a mild ocular phenotype. Craniofacial features associated with ARS include a prominent forehead, hypertelorism, maxillary hypoplasia, and a flattened mid-face with a broad, flat nasal bridge, thin upper lip, and protruding lower lip. Dental anomalies include microdontia (small teeth), cone-shaped teeth, or fewer teeth than normal [7, 8]. Additional noncommon features of ARS include pituitary abnormalities, hearing loss, kidney abnormalities, cardiovascular outflow tract malformation, and variable neurological and skeletal anomalies.

Disease heterogeneity has been generally recognized as the important challenge in diagnosis of ARS. The underlying genetic defects of ARS in 40% of patients is pathogenic variants in forkhead box C1 (*FOXC1*, MIM 601,090) or pituitary homeobox 2 *(PITX2*, MIM 601,542) [9, 10], which both have important roles in embryonic development and regulate downstream genes which are important for cell differentiation and migration via DNA binding [11]. *FOXC1* belongs to the FOX protein family, which are characterized by a winged helix DNA-binding domain [12]. Pathogenic variants in *FOXC1* correlate with anterior segment dysgenesis 3 and ARS.

To date, there have been a few cases of *FOXC1*-associated ARS described in the Chinese population [13–15]. We report on one 5-year-old Chinese boy with hypertelorism, conductive hearing loss, dental defects and pupillary deformation, not the core features of ARS and identified heterozygosity for a de novo deletion in the *FOXC1* gene by whole exome sequencing. This young boy was diagnosed with ARS via identification of this de novo variant in *FOXC1*. We also reviewed the literatures for cases of *FOXC1*-associated ARS.

## Materials and methods

### Clinical evaluation

A 5-year-old boy was admitted to the Department of Otolaryngology, the PLA Rocket Force Characteristic Medical Center (Beijing, China). His parents complained hearing loss, small teeth and hypertelorism of this affected boy. The study was approved by the PLA Rocket Force Characteristic Medical Center Ethics Committees. Clinical information was gathered through multiple interviews. Physical examination, otoscopy, pure tone audiometric examination, best corrected visual acuity (BCVA), slit-lamp examination, intraocular pressure (IOP) measurement and fundus examination by indirect ophthalmoscopy were performed on the patient. Cardiac structure and function were evaluated by ECG and color Doppler echocardiography. In addition, serum chemistry analysis, blood count, urinalysis, and chest X-ray were performed in the patient.

### Whole exome sequencing (WES) and bioinformatic analysis

WES genetic analysis were designed on offspring-parents trios, including one affected individual (II:1) and two unaffected parents (I:1 and I:2). Genomic DNA was extracted from peripheral blood using a blood DNA extraction kit according to the manufacturer’s instructions (TianGen, Beijing, China). DNA was sheared, ligated to adaptors, extracted, amplified by ligation-mediated PCR. 1 μg DNA library was mixed with Buffer BL and GenCap probe (MyGenostics, Beijing, China) for enrichment. Each captured library was loaded onto the Illumina NovaSeq 6000 platform. After filtering out low-quality and duplicate reads, clean data were aligned to the human reference genome hg19 using the Burrows-Wheeler Aligner. After alignment, variants were called using four types of software (SOAPsnp, GATK, Samtools and Platypus), merged into variant call format files and annotated by ANNOVAR and associated with multiple databases, including those with minor allele frequencies (MAF < 0.05), in public databases, including gnomAD, Inhouse database (MyGenostics), HGMD, and predicted by SIFT, PolyPhen-2, MutationTaster, GERP +  + . Under the assumption of an autosomal-recessive or autosomal-dominant (de novo) pattern of inheritance, only variants that were homozygous or compound heterozygous in the affected boy and heterozygous in their parents, and de novo variant were selected as candidates. VarSome was used for a comprehensive interpretation of the variants [16]. Manually classification of those variants was conducted based on American College of Medical Genetics and Genomics (ACMG)/Association for Molecular Pathology (AMP) guidelines for genetic hearing loss [17]. Potential pathogenic variants finally screened by these analyses were confirmed using Sanger sequencing.

### Multiple sequence alignment

Multiple sequence alignment was performed according to a Homologene program with default settings and the sequences *NP_001444.2* (*H.sapiens*), *XP_003311061.2* (*P.troglodytes*), *NP_032618.2* (*M. musculus*),* NP_001102359.1* (*R. norvegicus*), *NP_571803.1* (*X. tropicalis*) and *NP_001007864.1* (*D. rerio*).

(https://www.ncbi.nlm.nih.gov/homologene?cmd=Retrieve&dopt=MultipleAlignment&list_uids=20373).

## Results

### Clinical evaluations

**Fig. 1:**
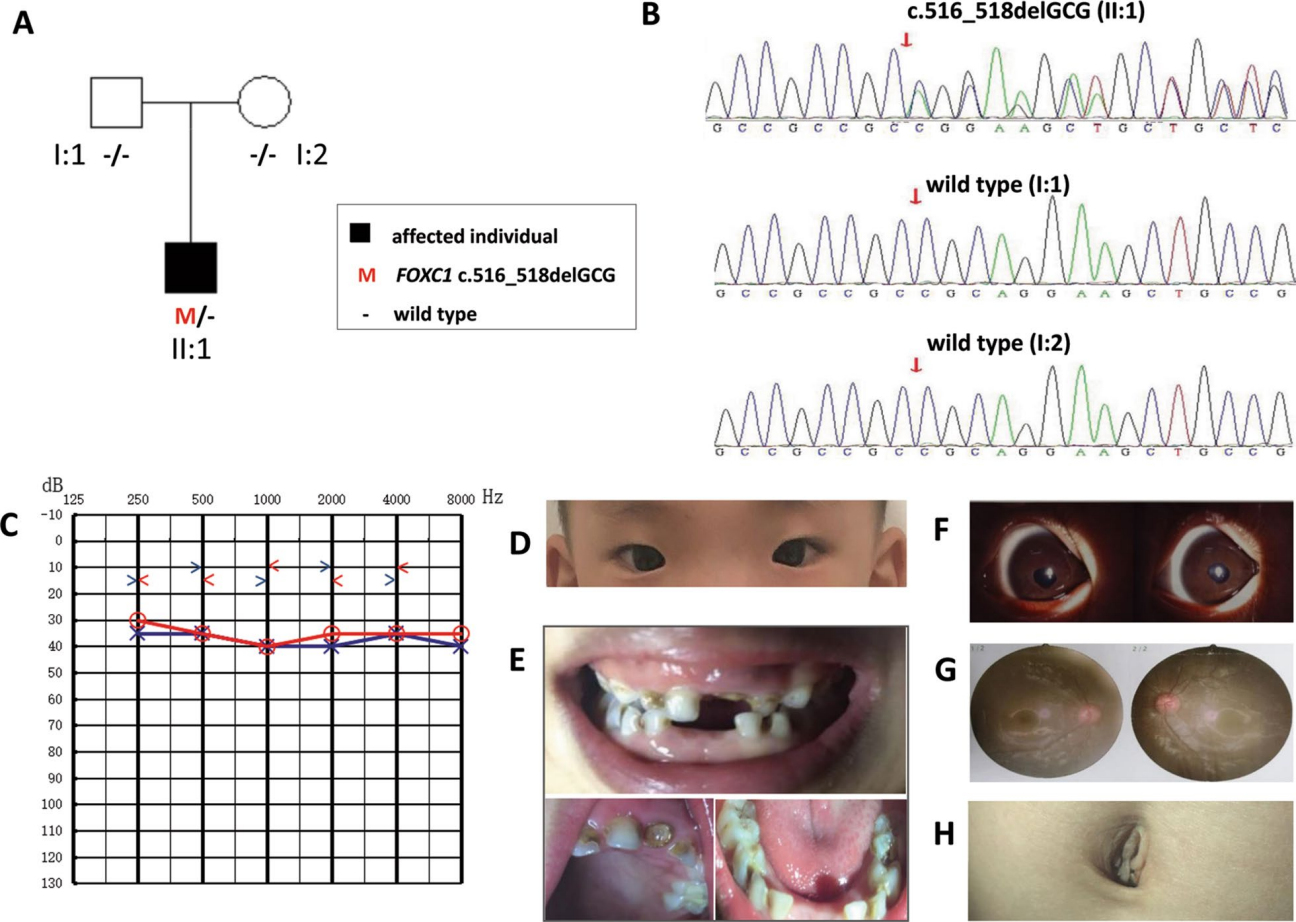
Pedigree, variant analysis and systemic anomalies of the patient (II:1). **a** The proband is indicated by an arrow. Subject II:1, I:1 and I:2 were tested by WES. **b** Chromatogram shows *FOXC1* heterozygous c.516_518delGCG detected in patient II:1. **c** Audiogram showed bilateral conductive hearing loss of affected subjects II:1 (red, right ear; blue, left ear). **d** Hypertelorism of the patient. **e** Microdontia (small teeth), cone-shaped teeth, and fewer teeth of the patient. **f** Slit-lamp examination revealed pupil deformation in both eyes. **g** The ocular fundus was normal and the cup-to disc ratio in each eye was about 0.3. **h** Normal periumblical skin of the patient II:1

We have clinically examined one affected boy (II:1) and his unaffected parents from this family (I:1 and I:2). The family pedigree is presented in Fig. 1a, and a summary of the main clinical characteristics is presented in comparison of those cases reported thus far (Table 1).

**Table 1:** Hearing phenotypes associated with *FOXC1* pathogenic variants

*FOXC1* variants	Hearing phenotype	Types	Age of onset	Number of ear affected	Severity	Progression	Frequency affected
c.26–47ins(p.Ser9fs*89)	Hearing loss	N/A	N/A	N/A	N/A	N/A	N/A
c.67C > T (p.Gln23*)	Hearing loss	N/A	N/A	N/A	N/A	N/A	N/A
c.210delG(p.Gln70fs*73)	Hearing loss	N/A	N/A	N/A	N/A	N/A	N/A
c.235C > A(p.Pro79Thr)	Hearing loss	N/A	N/A	N/A	N/A	N/A	N/A
c.245G > C (p.Ser82Thr)	Hearing loss	N/A	N/A	N/A	N/A	N/A	N/A
c.317delA (p.Gln106Argfs*75)	Normal hearing	−	−	−	−	−	−
c.325A > G(p.Met109Val)	Hearing loss	N/A	N/A	N/A	N/A	N/A	N/A
c.335del(p.Phe112Serfs*69)	Hearing loss	N/A	N/A	Unilateral	N/A	N/A	N/A
c.339 T > C (p.Tyr115Ser)	Hearing loss	Conductive	N/A	N/A	N/A	N/A	N/A
c.344A > C(p.Tyr115Ser)	Hearing loss	Conductive	N/A	N/A	N/A	N/A	N/A
c.437_453del17(p.Pro146fs)	Hearing loss	N/A	N/A	N/A	N/A	N/A	N/A
c.454 T > C(p.Trp152Arg)	Hearing loss	N/A	N/A	N/A	Mild	N/A	N/A
c.457A > C (p.Thr153Pro)	Hearing loss	N/A	N/A	N/A	N/A	N/A	N/A
c.477C > G(p.Tyr159*)	Hearing loss	Sensorineural	N/A	Bilateral	N/A	Progressive	N/A
c.478_482dupAACAT(p.Met161Ilefs*22)	Hearing loss	Sensorineural	N/A	N/A	N/A	N/A	N/A
c.481A > G(p.Met161Val)	Hearing loss	Conductive	N/A	N/A	N/A	N/A	N/A
c.487G > T(p.Glu163*)	Hearing loss	N/A	N/A	N/A	N/A	N/A	N/A
c.506G > C(p.Arg169Pro)	Hearing loss	N/A	N/A	N/A	N/A	N/A	N/A
c.508C > T(p.Arg170Trp)	Hearing loss	N/A	N/A	N/A	N/A	N/A	N/A
**c.516_518delGCG (p.Arg173del)**	**Hearing loss**	**Conductive**	**5 yo**	**Bilateral**	**Mild**	**Reversible**	**ALL frequencies**
c.719delT(p.Leu240Argfs*75)	Hearing loss	N/A	N/A	N/A	N/A	N/A	N/A
c.1193_1196dup(p.Met400Serfs*129)	Hearing loss	N/A	Congenital	N/A	N/A	N/A	N/A
c.1491C > G (p.Tyr497*)	Hearing loss	N/A	N/A	N/A	N/A	N/A	N/A
deletion incl entire gene & GMDS	Hearing loss	N/A	N/A	N/A	N/A	N/A	N/A
deletion 6,610 kb incl. entire gene	Hearing loss	Sensorineural	N/A	Bilateral	N/A	N/A	N/A
Translocation t(6;8)(p25;q24.1)	Hearing loss	N/A	N/A	N/A	Mild	N/A	N/A
Deletion ~ 12 Mb incl entire gene, FOXQ, FOXF2 & others	Hearing loss	N/A	N/A	N/A	Severe	N/A	N/A
deletion 2.2–2.4 Mb incl entire gene	Hearing loss	Mixed	N/A	N/A	N/A	N/A	N/A
deletion 4.7 Mb incl. entire gene	Hearing loss	Conductive(due to chronic glue ear)	N/A	N/A	N/A	N/A	N/A
1.3 Mb del incl gene & 3 others, 0.52 Mb dup incl 13 genes	Hearing loss	N/A	N/A	N/A	N/A	N/A	N/A
1.10 Mb del incl entire gene, GMDS & part of LOC340156	Hearing loss	N/A	N/A	N/A	N/A	N/A	N/A
0.98 Mb deletion incl entire gene & GMDS	Hearing loss	N/A	N/A	N/A	N/A	N/A	N/A
Deletion 34 kb incl. entire gene(1,551,415–1,585,522)	Hearing loss	N/A	N/A	N/A	N/A	Progressive	N/A
Translocation t(2;6)(p32;q24)	Hearing loss	N/A	N/A	N/A	N/A	Progressive	N/A
Deletion 3.4 Mb incl. entire gene(566,884–3,960,186)	Hearing loss	Conductive	N/A	N/A	N/A	N/A	N/A
Deletion ~ 2.7 Mb incl entire gene & 9 others(6p25.2-pter)	Hearing loss	Sensorineural	N/A	N/A	Mild	N/A	N/A
Deletion 5.0–5.7 Mb incl. entire gene	Hearing loss	Sensorineural	N/A	Bilateral	N/A	N/A	N/A
Deletion 6.57 Mb (6p25.1-pter)	Hearing loss	Sensorineural	N/A	Bilateral	Mild to moderate	N/A	Low-frequency
Deletion 2.6 Mb incl. entire gene(0–2,646,377)	Hearing loss	Conductive(due to middle ear malformations)	N/A	N/A	N/A	N/A	N/A
Deletion ~ 8 Mb incl entire gene, FOXQ, FOXF2 & others	Hearing loss	N/A	N/A	N/A	Severe	N/A	N/A
1.5 Mb deletion	Normal hearing	−	−	−	−	−	−
6p25-6pter deletion	Normal hearing	−	−	−	−	−	−
3.9 Mb deletion	Normal hearing	−	−	−	−	−	−

yo, years old. -, not applicable. N/A, not assessed. Marked in bold, variant identified in this study

Detailed ocular analysis and auditory function was performed in the affected boy II:1. Although sensorineural hearing loss has been usually reported in *FOXC1*–related disease, conductive hearing loss induced by otitis media with effusion was identified in this patient, which is in accordance with observation of the pure tone hearing test, temporal bone CT and hearing recovery after 3 months (Fig. 1c). Craniofacial features observed in the proband include midface hypoplasia, hypertelorism (Fig. 1d). Dental examination showed microdontia (small teeth), cone-shaped teeth, and fewer teeth than normal (Fig. 1e). The best-corrected visual acuity was 20/20 in both eyes (OU). The intraocular pressure (IOP) was normal, of which 11 mmHg in the right eye and 10 mmHg in the left eye. Slit-lamp examination revealed pupillary deformation (corectopia) in OU (Fig. 1f). The ocular fundus was normal and the cup-to disc ratio in each eye was about 0.3 (Fig. 1g). The umbilicus was normal (Fig. 1h). No heart defects, pituitary abnormalities, kidney abnormalities, neurological and skeletal anomalies were observed. This affected individual did not have obvious delayed gross motor development. The proband was diagnosed with ARS based on the ocular and systemic signs.

### Genotyping

**Fig. 2:**
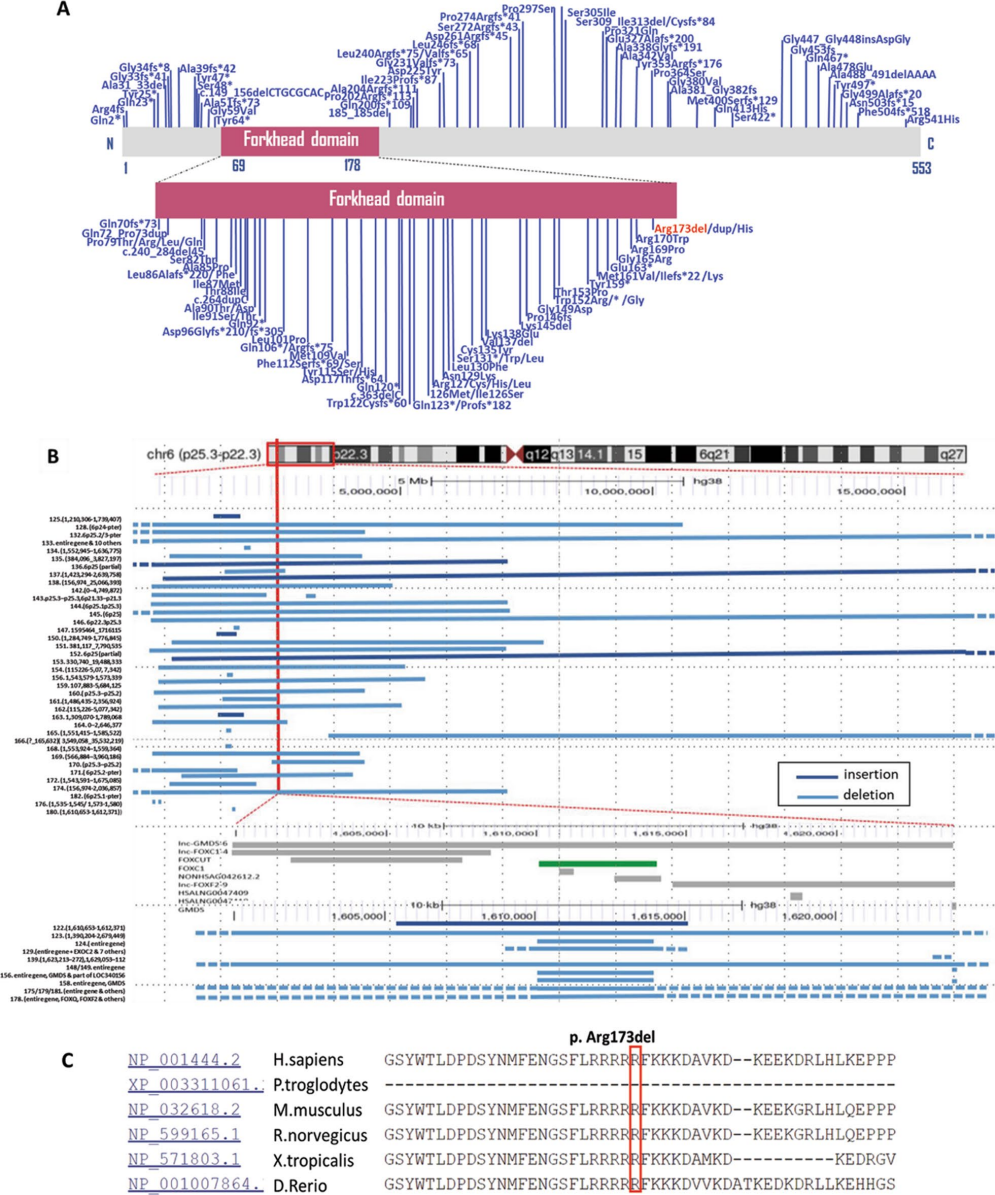
Overview of reported pathogenic variants in *FOXC1* (NP_001444.2). **a** Locations of single nucleotide variants and Indels, including missense/ nonsense, splicing, small deletion/ small insertions. The position of *FOXC1* p.Arg173del is highlighted in red. **b** Location of genomic structural variants including gross deletions/ insertions. The p25.3-p22.3 region of interest is surrounded by a red box on the chromosome 6 ideogram, and the chromosome 6 p25.3 is shown as a red line, which includes the *FOXC1* gene and other gene loci. Such as: *GMDS*, *FOXCUT*, *FOXF2*, etc. The dashed line represents the fragment beyond the range of p25.3-p22.3, the green bar represents the target gene *FOXC1*, and the gray bar represents its surrounding genes. **c** Conservational analysis of *FOXC1* p.Arg173del. Protein alignment showing *FOXC1* p.Arg173del occurred at evolutionarily conserved amino acids (in red box) across 6 species

The average depth of the sequencing data was 103.24, the average coverage for data was 99.91%, the fraction of target regions covered with at least 10 × and 20 × was 98.96% and 96.76%, respectively. Variants remaining after filtering were manually assessed based on frequency / presence in known SNP databases, any previous association with disease, predicted functional impact, any nucleotide / amino acid conservation, and the potential detrimental biochemistry of any amino acid substitution observed. Using Sanger sequencing, three participating family members (one affected and two unaffected) in this family were genotyped to identify the candidate variants. One de novo variant c.516_518delGCG (p.Arg173del) in *FOXC1* (NM_001453.3) and two compound heterozygous variants in *ERCC6* (NM_000124), c.2444G > A (p.Gly815Asp) and c.2125G > A (p.Val709Ile) remained after the filtering process. *FOXC1* c.516_518delGCG (p.Arg173del) deletion occurred in an evolutionarily highly conserved region across different species in the forkhead domain (Fig. 2), resulted in one amino acid Arginine deletion at position 173. This variant has not been reported previously, nor found in the gnomAD. Recently, Ma et al*.* reported two novel variants in *FOXC1* affecting p.Arg173, one missense variant c.518G > A, p.(Arg173His) and one in-frame duplication c.516_518dupGCG p.(Arg173dup), in two unrelated patients with Axenfeld–Rieger anomaly, both presented with posterior embryotoxon and evidence of iris adhesions. However, no dental defects, craniofacial dysmorphism and hearing loss were described in these two patients [18]. *FOXC1* heterozygous variant c.516_518delGCG (p.Arg173del) was only found in the patient II:1 and was absent in his unaffected parents (Fig. 1b), consistent with autosomal dominant inheritance (*de nov*o). Pathogenic computational verdict based on 1 pathogenic prediction from phyloP vs no benign prediction. According to the American College of Medical Genetics and Genomics (ACMG), c.516_518delGCG (p.Arg173del) is classified as a pathogenic variant (PS2 + PM1 + PM2 + PP3 + PP4) [16, 17, 19].

Two variants in *ERCC6,* c.2444G > A (p.Gly815Asp) and c.2125G > A (p.Val709Ile) were both classified as variants with uncertain significance. *ERCC6* encodes a DNA-binding protein that is important in transcription-coupled excision repair. Pathogenic variants in *ERCC6* are associated with Cockayne Syndrome, which is an autosomal recessive disorder and characterized by postnatal developmental failure, progressive neurodegeneration, microcephaly, and premature aging. These phenotypes were not in accordance with the proband’s clinical presentations.

These data, together with the clinical presentation of the patient and consistent autosomal dominant inheritance of the *FOXC1* gene, indicate that *FOXC1* heterozygous variant c.516_518delGCG (p.Arg173del) is the cause of ARS in the proband.

## Discussion

The *FOXC1* gene (MIM #601090) encodes a transcription factor, FOXC1, which is characterized by a highly conserved approximately 110-amino-acid motif (69-178aa) known as the forkhead domain. The nuclear localization signals at either end of the forkhead domain support the transfer of FOXC1 to the nucleus and biding to DNA, thereby regulating the downstream target genes. FOXC1 is highly expressed in the heart, kidney and skeletal muscles, and has a role in tumor development, tissue-specific gene expression and embryogenesis. According to HGMD, 181 different pathogenic variants of *FOXC1* have been identified, which are related with ARS and/or ASD (Anterior segment dysgenesis) (Additional file 1: Table 1, Fig. 2). Most pathogenic variants affect the amino acid within the forkhead domain, which impair the FOXC1 protein function by altering the ability of nuclear localization, transactivation activity, DNA-biding ability and protein stability [20, 21]. Pathogenic variants at the C-terminal of *FOXC1* are less characterized, probably influences FOXC1 protein ubiquitination [13].

Based on genetic analyses combined with ocular and the systemic abnormalities including craniofacial dysmorphism and dental malformation, we made a diagnosis of ARS in the proband [1]. Structure of the anterior segment was affected which is characterized by pupil deformity in both eyes. Apart from pupil deformity (corectopia), patients with ARS may have various anterior segment abnormalities such as posterior embryotoxon, iris stromal hypoplasia, pseudopolycoria and angle closure [22, 23]. Among them, destruction of the chamber angle often leads to secondary glaucoma. Patients may develop into glaucoma at a very young age, or they may be asymptomatic for a long time from childhood to youth until high intraocular pressure occurs. It is reported that more than 50% patients with *FOXC1*-related ARS will develop into glaucoma eventually and the median onset age of glaucoma diagnosis is 6 ± 13.0 years [3, 24, 25]. In this study, although the 5-year-old boy had normal intraocular pressure and there were no alterations of the optic nerve, he is still at high risk of developing glaucoma. Due to the high risk and adverse consequence, it is crucial for the patients with ARS to follow up frequently and take a comprehensive ophthalmic examination. IOP monitoring, anterior chamber angle examination and fundus examination are necessary for follow-up. Automated perimetry and optic nerve head optical coherence tomography should be further improved to evaluate visual field defect and optic nerve injury in patients who are suspected to suffer from glaucoma. Early diagnosis and treatment are crucial to control the ocular complications of ARS and reduce the incidence of blindness caused by glaucoma. At present, surgical treatment is applied to decrease the raised IOP which correlates well with glaucoma progression and leads to satisfactory visual outcome in a long term [26].

Hearing loss is an infrequently characterized feature in ARS patients, with sensorineural hearing loss being the most prevalent type [27]. However, conductive hearing loss have been reported in several literatures (Table 1). Also, structural abnormalities of the cochlea have been observed in a few individuals. Although a precise mechanism for FOXC1 in hearing or ear development is not well understood. By systematic review of hearing phenotypes associated with *FOXC1* pathogenic variants, 38 variants were reported to be related with abnormal hearing (Table 1), including 11 variants caused either a frameshift or premature stop codon and 17 gross deletions/insertions/complex rearrangements, leading to the total or partial loss of the FOXC1 function. Only one frameshift variant within the forkhead domain (p.Gln106Argfs*7) and 3 gross deletions reported normal hearing (Table 1) [28]. There are, however, many patients with *FOXC1* variant involving the forkhead domain that do not report hearing problems. This observation may be due to variable expressivity of the hearing phenotype, or could implicate a second gene or other factors that impact hearing pathogenesis. Moreover, in many patients, there is no mention of the hearing phenotype, making it difficult to determine whether or not hearing tests were conducted. Although the proband in our study presented hearing loss, however, by detailed hearing evaluation, the diagnosis of conductive hearing loss induced by otitis media with effusion was reached. Hearing recovery and normal hearing was observed 3 months later, indicating that the hearing loss is reversible and coincidental and it might not linke to the *FOXC1* variant.

Minor facial dysmorphism has been identified as part of the ARS spectrum since the initial reports of affected individuals, with characteristic facial features including maxillary hypoplasia with flattening of the midface, hypertelorism, a broad flat nasal root, and a thin upper lip [29]. Hypertelorism is typically a manifestation of a craniofacial deformity and not a disease in itself. It can be seen in a variety of conditions such as craniofacial cleft, craniofacial dysplasia and craniosynostosis syndromes, like Edwards Syndrome, 1q21.1 Duplication Syndrome, Basal Cell Nevus Syndrome, DiGeorge Syndrome Loeys-Dietz Syndrome, Apert Syndrome, Noonan syndrome, Neurofibromatosis, Leopard Syndrome, Crouzon Syndrome, Wolf-Hirschhorn Syndrome, Andersen–Tawil Syndrome, Waardenburg Syndrome and Cri-du-Chat Syndrome, et al. [30]. Assessment of dysmorphic features, including facial dysmorphism, plays a major role in the evaluation of genetic syndromes to reach a clinical diagnosis.

In summary, the present study described the clinical and genetic characteristics of a young Chinese boy with atypical ARS caused by a novel pathogenic variant in *FOXC1*, c.516_518delGCG (p.Arg173del), who was successfully diagnosed by whole exome sequencing (WES). This 3-bp deletion caused one Arginine loss, in the forkhead domain, might disrupt the secondary structure of the FOXC1 protein. Generally, children with *FOXC1* variants are prone to developing into glaucoma and other ocular disorder, which underline the necessity of definite diagnosis by genetic methods along with subsequent early prevention of ocular defects. Regular monitoring concerning potential complications is required in ARS patients. The present results broadened the spectrum of *FOXC1* variants in patients with ARS, especially within patients with atypical ARS.

## Supplementary Information

**Additional file 1.** Summary of clinical features and molecular results in patients with FOXC1 pathogenic variants reported in HGMD and this study.

## Data Availability

The patient’s phenotype and the detected variants have been submitted to the ClinVar (https://www.ncbi.nlm.nih.gov/clinvar/) and the submission accession number is SUB10323251.

## Abbreviations

ARS: Axenfeld-Rieger syndrome
FOXC1: Forkhead box C1
PITX2: Pituitary homeobox 2
BCVA: Best corrected visual acuity
IOP: Intraocular pressure
WES: Whole exome sequencing
ACMG: American college of medical genetics and genomics
AMP: Association for molecular pathology
ASD: Anterior segment dysgenesis
